# Flapless Guided Dental Implant Surgery in a Systemically Compromised Patient: A Case Report

**DOI:** 10.7759/cureus.67582

**Published:** 2024-08-23

**Authors:** Pamela Campos, Rafael Manfro, Gustavo Coura, Márcio de Carvalho Formiga

**Affiliations:** 1 Oral Implantology, Univali São José, São José, BRA

**Keywords:** bone density, implant drills, 3d printing, dental implants, guided surgery

## Abstract

The use of surgical guides in implant treatment offers several advantages, including reduced surgery time, flapless procedures, and enhanced precision and reliability in implant placement. By utilizing tomography and scanning technologies, a patient’s complete anatomical structure can be transferred into software in three spatial planes. This allows for the three-dimensional printing of surgical guides that ensure the accurate execution of the procedure. Traditional techniques and planning using two-dimensional imaging are now considered outdated compared to the precision offered by guided surgery. In this study, we present the case of a patient with multiple systemic health complications whose primary concern was the absence of teeth 15 and 35. For this patient, flap surgeries were contraindicated. Given the effectiveness of surgical guides, this method was chosen for planning and treatment. Single Implacil implants were successfully placed. Despite the limitations of this case report, the use of guided surgery proved to be more precise and effective in determining the location, direction, and inclination of the implants while simultaneously enhancing patient comfort.

## Introduction

Initially, dental implants were placed with the sole aim of ensuring osseointegration. Over time, deviations from the correct implant position were compensated for during the prosthesis fabrication, leading to frequent errors due to improper positioning. Many researchers have highlighted the need for presurgical planning tools to improve accuracy in implant surgery and prosthetic rehabilitation [1,2]. Effective treatment requires collaboration between the surgeon and prosthetist, starting with the creation of a surgical guide to precisely locate each implant. Thus, achieving osseointegration alone is no longer adequate; three-dimensional (3D) positioning has become crucial to meeting patient needs for health, function, and aesthetics [3].

Initially, implant placement relied on X-rays and plaster models. Panoramic X-rays provided only bone height, not thickness, increasing the risk of failure [4]. With advancements in imaging and planning, relying solely on panoramic radiography is no longer sufficient, as two-dimensional imaging can result in inaccuracies and distortion [3].

Traditional implant protocols often involve longer surgery times, resulting in more postoperative scarring and discomfort for patients [5]. Guided surgery has emerged as a solution, utilizing devices that precisely direct the 3D positioning of implants. Custom surgical guides are developed for each patient, enabling accurate determination of implant direction, location, and inclination [6].

Flapless guided surgery offers several advantages, including reduced postoperative trauma and bleeding due to the precision of implant placement and the shorter surgical time. This technique employs specialized software to create 3D-printed surgical guides, minimizing distortions from the initial plan [5]. Turkyilmaz [7] noted that virtual planning and 3D printing of surgical guides lead to significantly greater accuracy compared to freehand techniques.

Guided surgery enhances planning and visualization for the implant dentist, improves communication between the dental surgeon and prosthodontist, and reduces patient morbidity [5]. The technology incorporates anatomical characteristics of the maxilla and mandible into the procedure, establishing a more effective protocol for functional and aesthetic rehabilitation [6].

The benefits of guided surgery include high predictability of results, reduced surgical time, minimized risk of injury to critical structures, less pain, faster healing, and decreased use of medications and anesthetics [8-10].

This study aims to report a clinical case involving a patient with comorbidities who underwent dental implantation using guided surgery and to highlight the advantages of this flapless technique compared to conventional methods.

## Case presentation

The patient, a 34-year-old woman, presented with an esthetic concern due to the absence of teeth 15 and 35. She had type I diabetes, but her endocrinologist approved her desire for dental implants with restrictions on corticosteroid use. Additionally, she had a history of cerebral thrombosis in 2015 and was using anticoagulants. Her hematologist advised discontinuing the medication three days before the surgical procedure. She had no known allergies to any products or medications.

**Figure 1:**
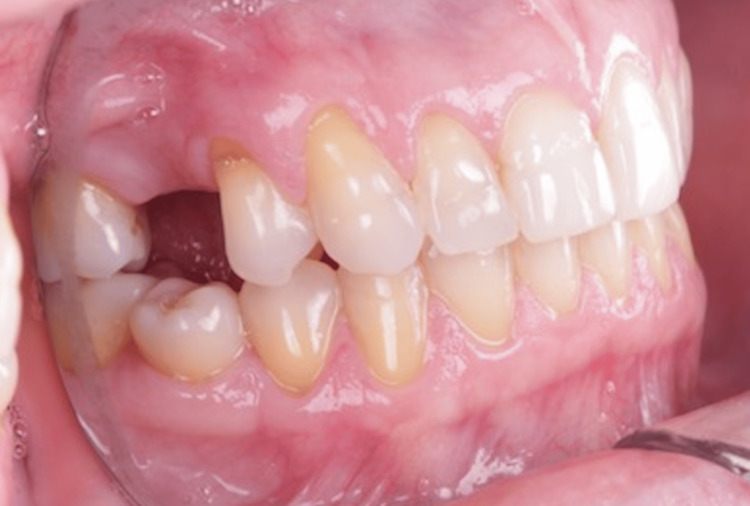
Right side view showing the absence of tooth 15

**Figure 2:**
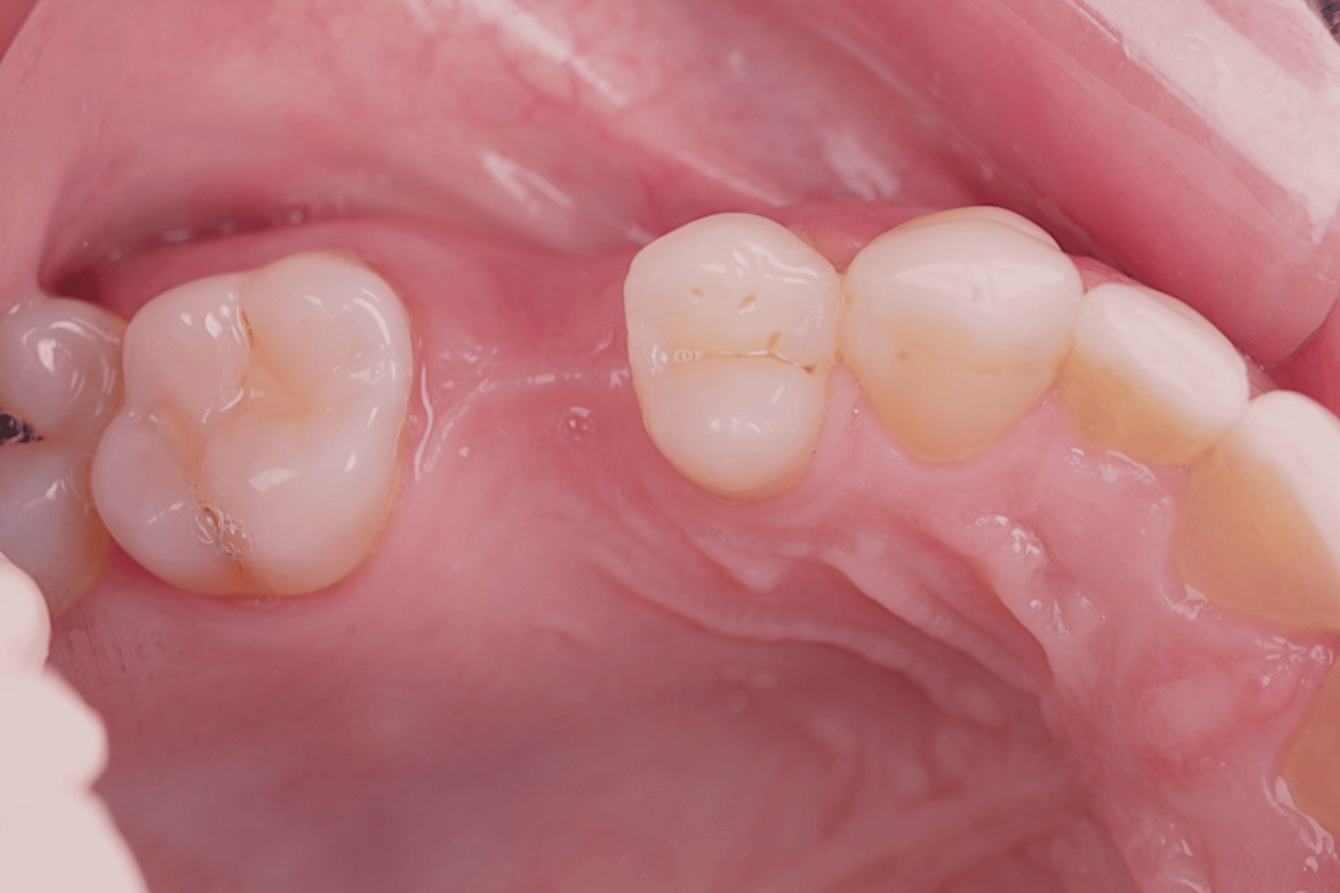
Occlusal side view showing the absence of tooth 15

**Figure 3:**
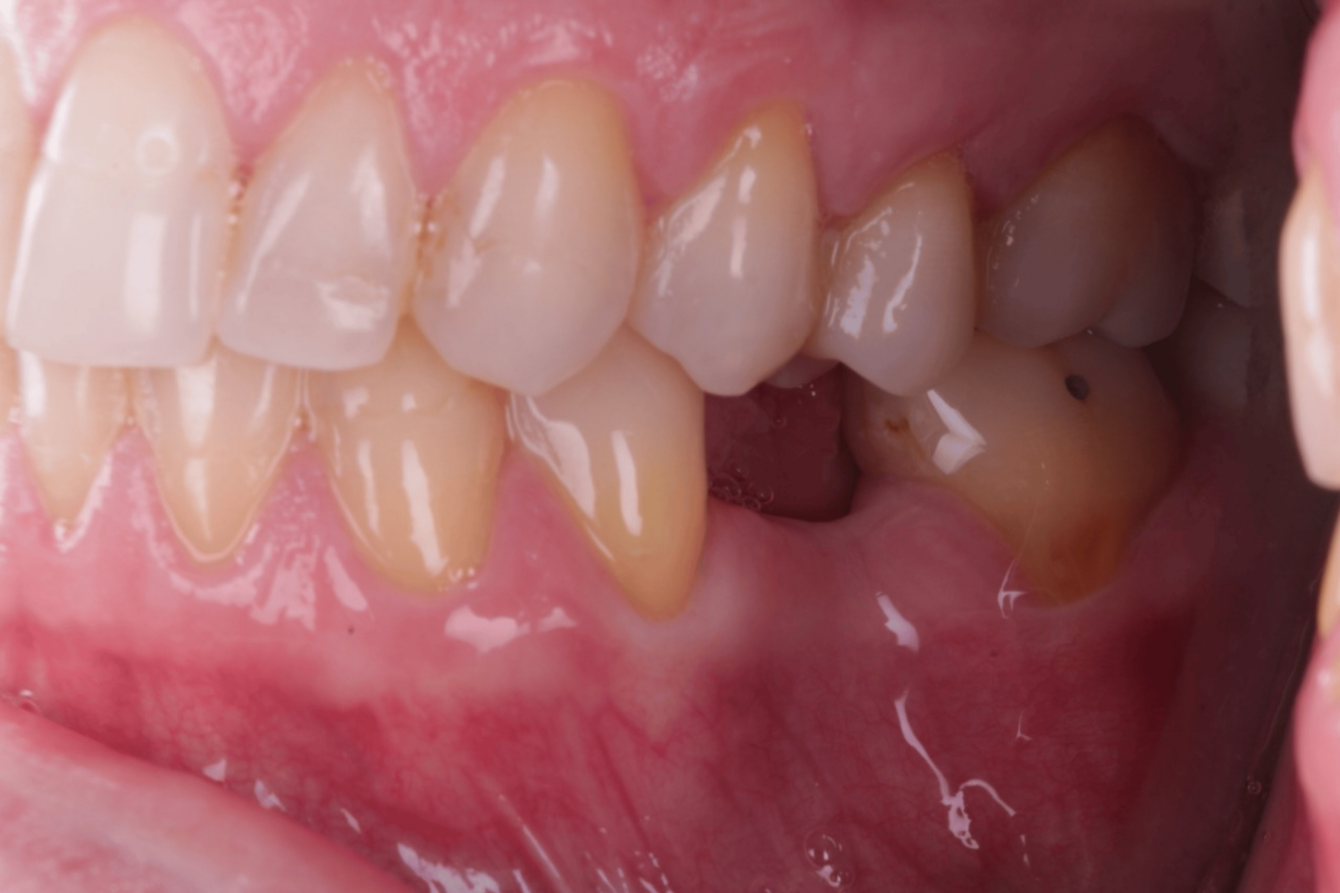
Left side view showing the absence of tooth 35

**Figure 4:**
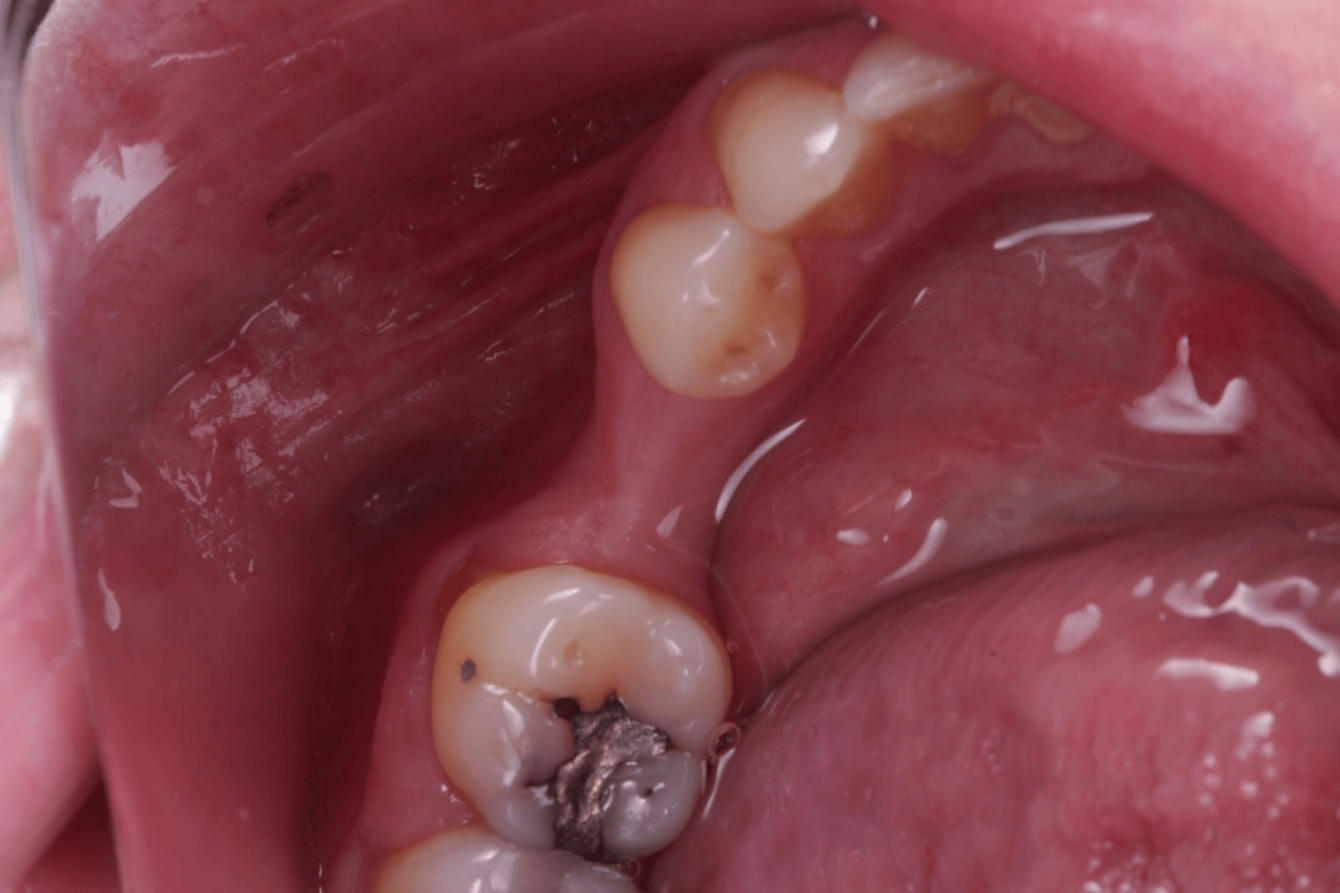
Left side occlusal view showing the absence of tooth 35

Her anamnesis was favorable, as she reported no issues with chewing, discomfort, or bleeding gums. An intraoral evaluation confirmed the absence of teeth 15 (Figure 1, Figure 2) and 35 (Figure 3, Figure 4).

**Figure 5:**
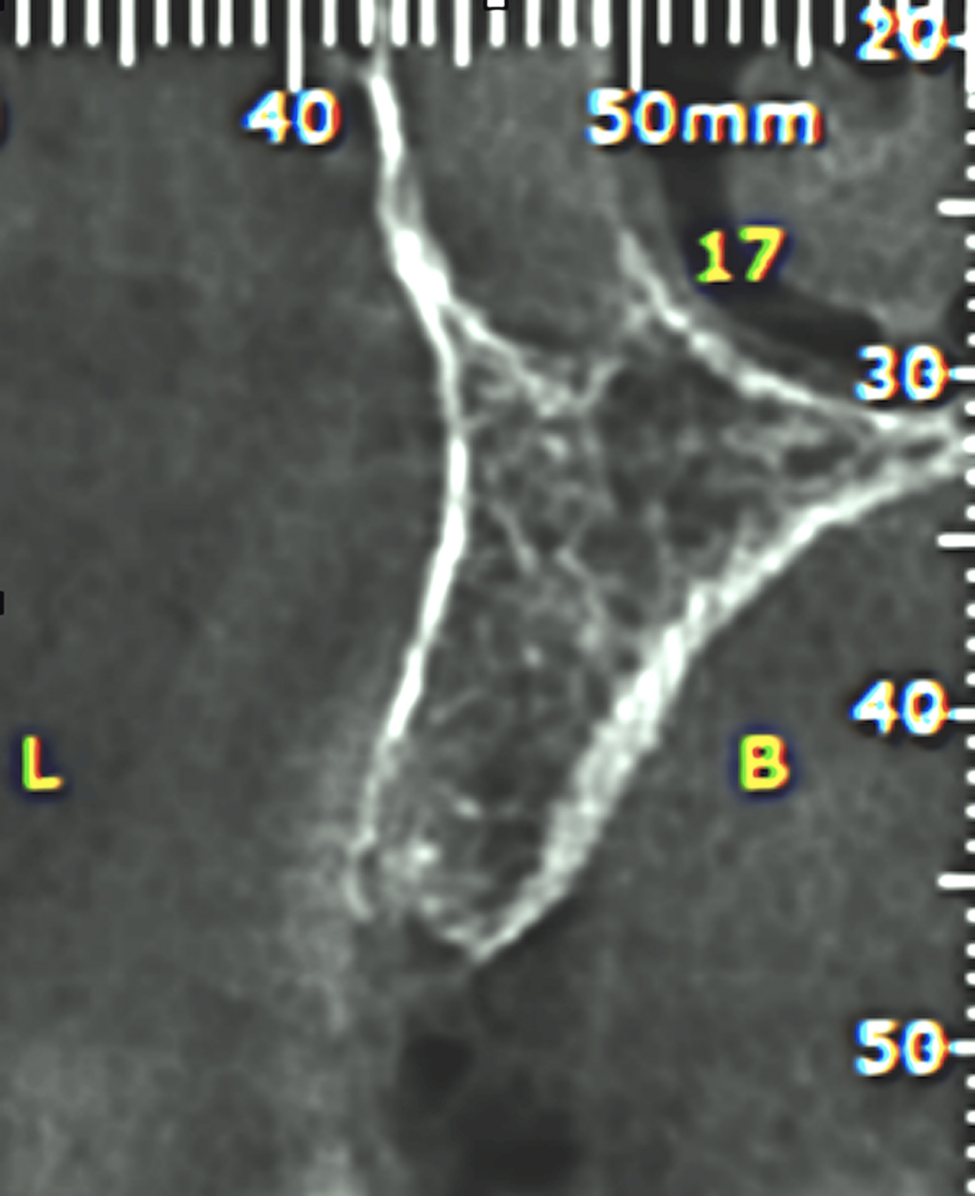
Baseline tomograph view of tooth 15, demonstrating adequate bone volume for implant placement

**Figure 6:**
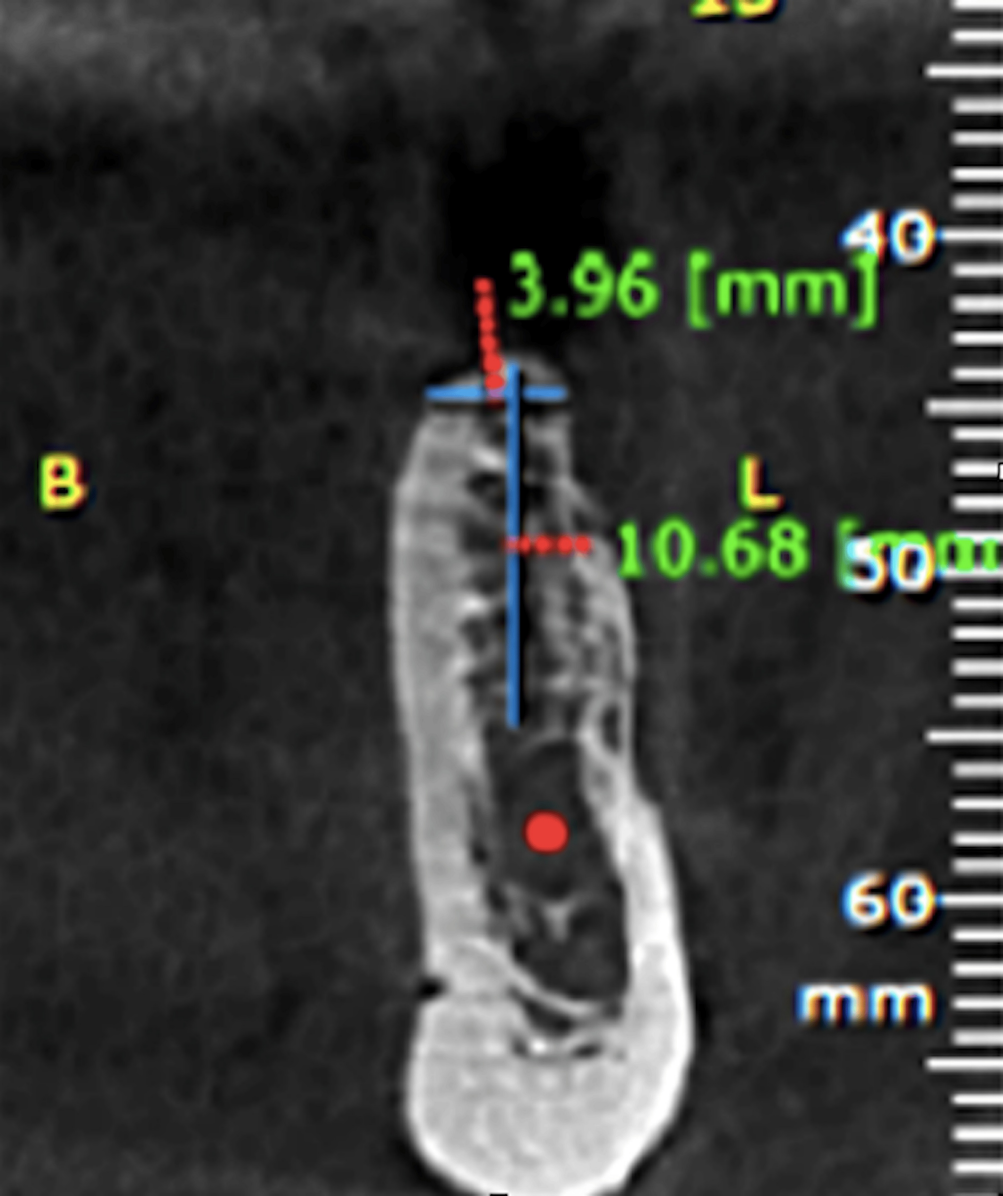
Baseline tomograph view of tooth 35, showing sufficient bone volume for implant placement

Tomography was performed to assess the quality and quantity of bone for implant placement (Figure 5, Figure 6).

**Figure 7:**
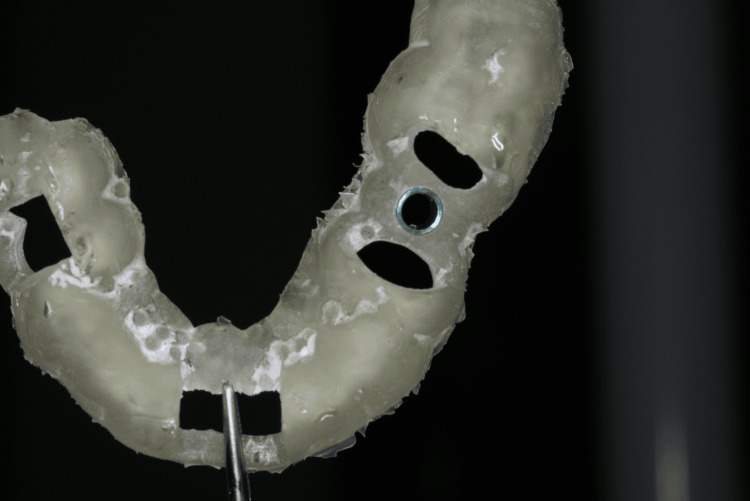
Printed surgical guide for tooth 15

Based on the Digital Imaging and Communications in Medicine (DICOM) images, a 3D surgical guide was planned and printed according to the digital design (Figure 7). Surgical guides were used for both teeth without flap elevation, given the patient’s systemic complications from diabetes and the associated risk of thrombosis.

**Figure 8:**
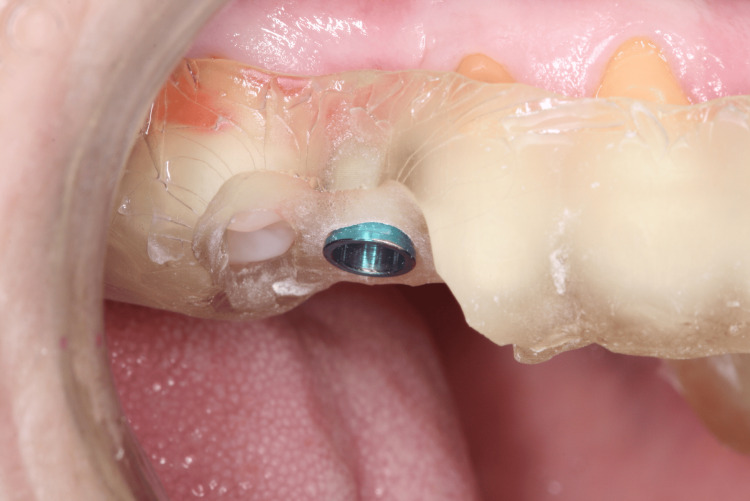
Right side view of tooth 15 showing the surgical guide in position

**Figure 9:**
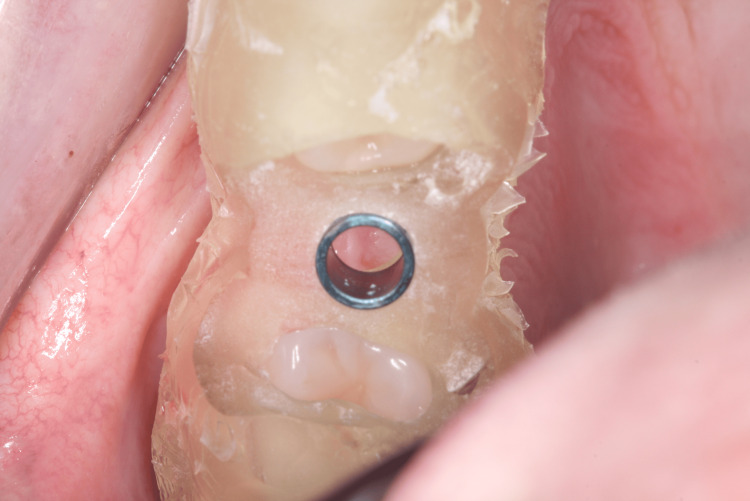
Right side occlusal view of tooth 15 showing the surgical guide in position

**Figure 10:**
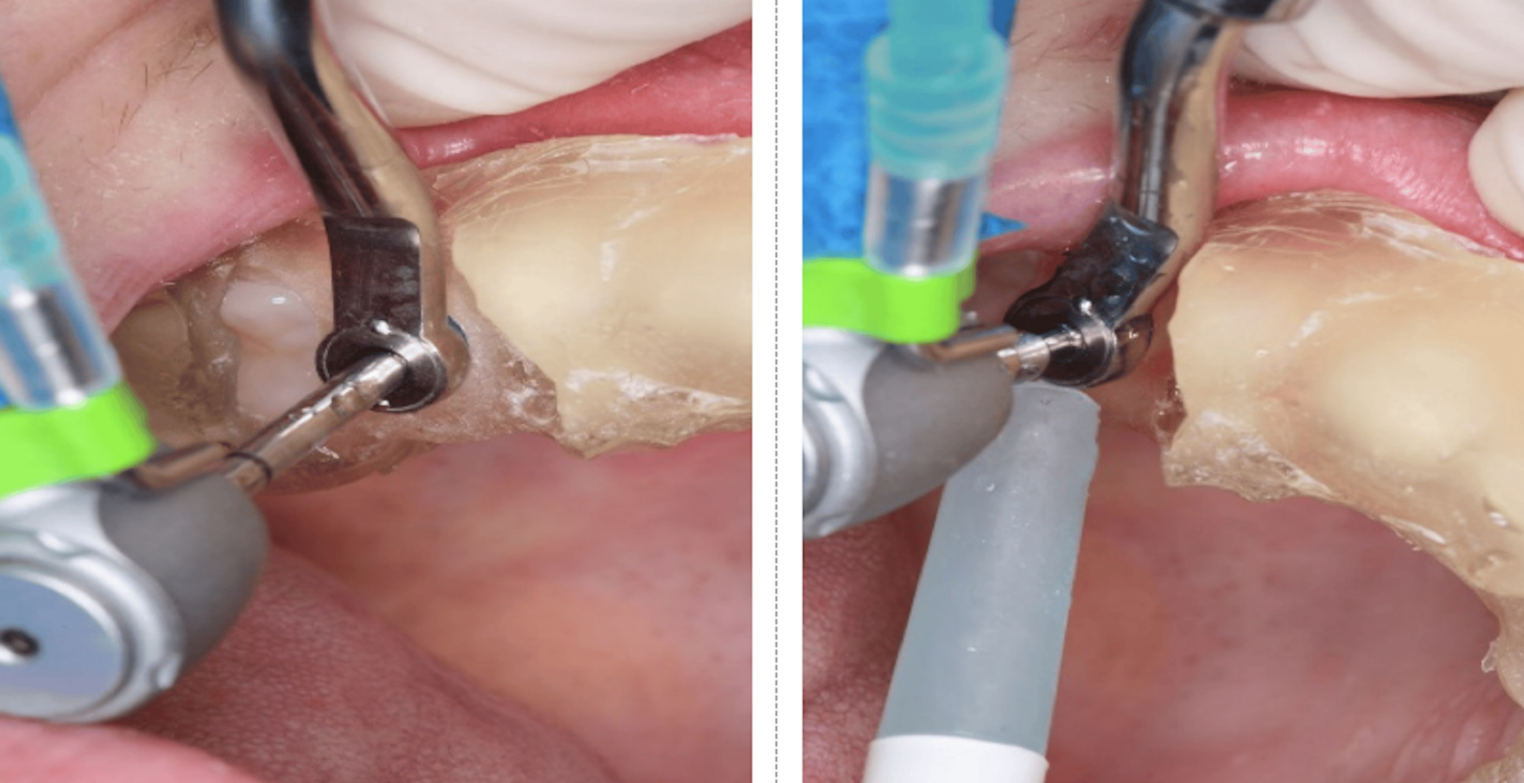
Use of Drill Spear 2.0 for initial drilling

**Figure 11:**
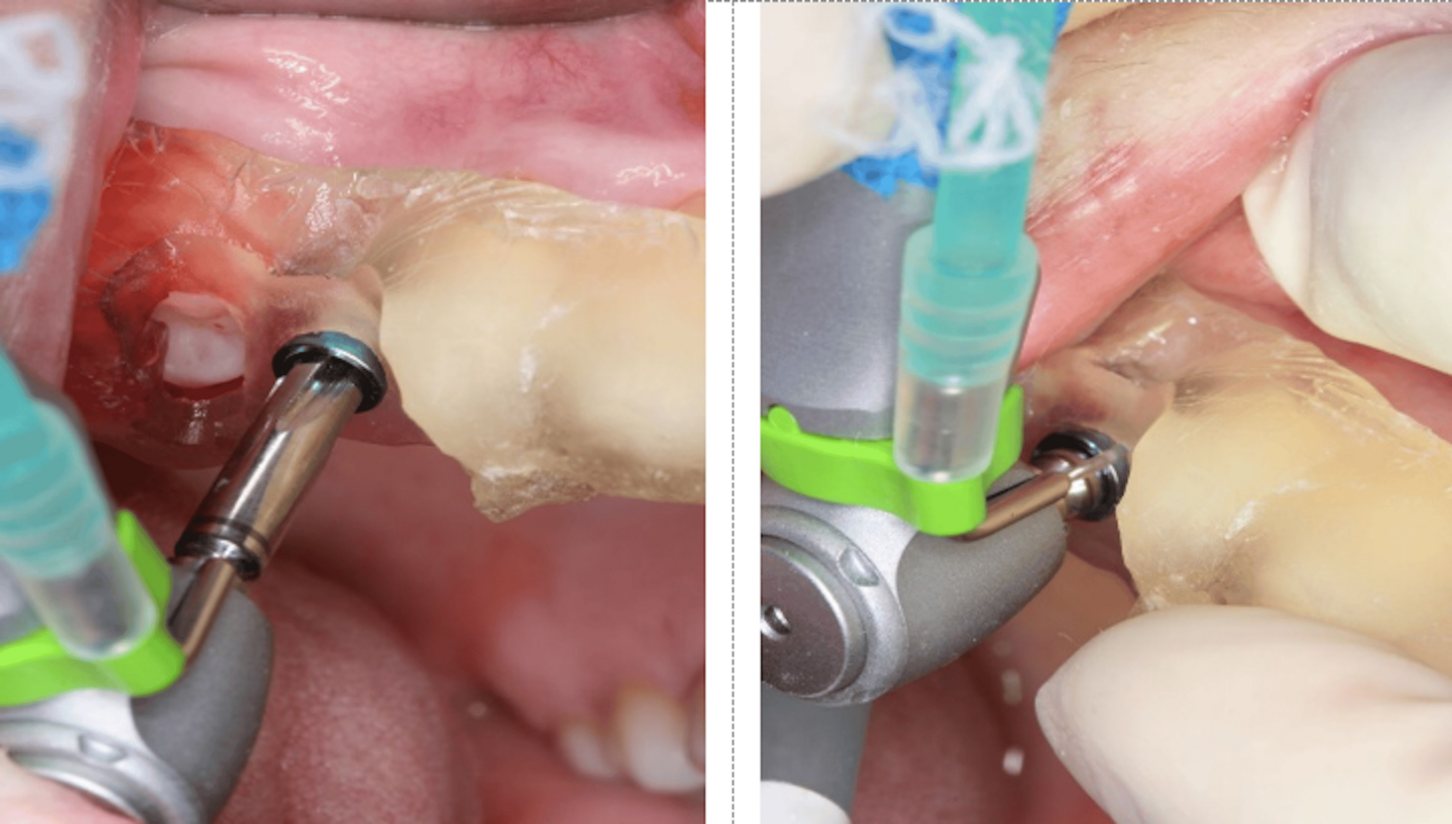
Instrumentation with Drill 2.8

**Figure 12:**
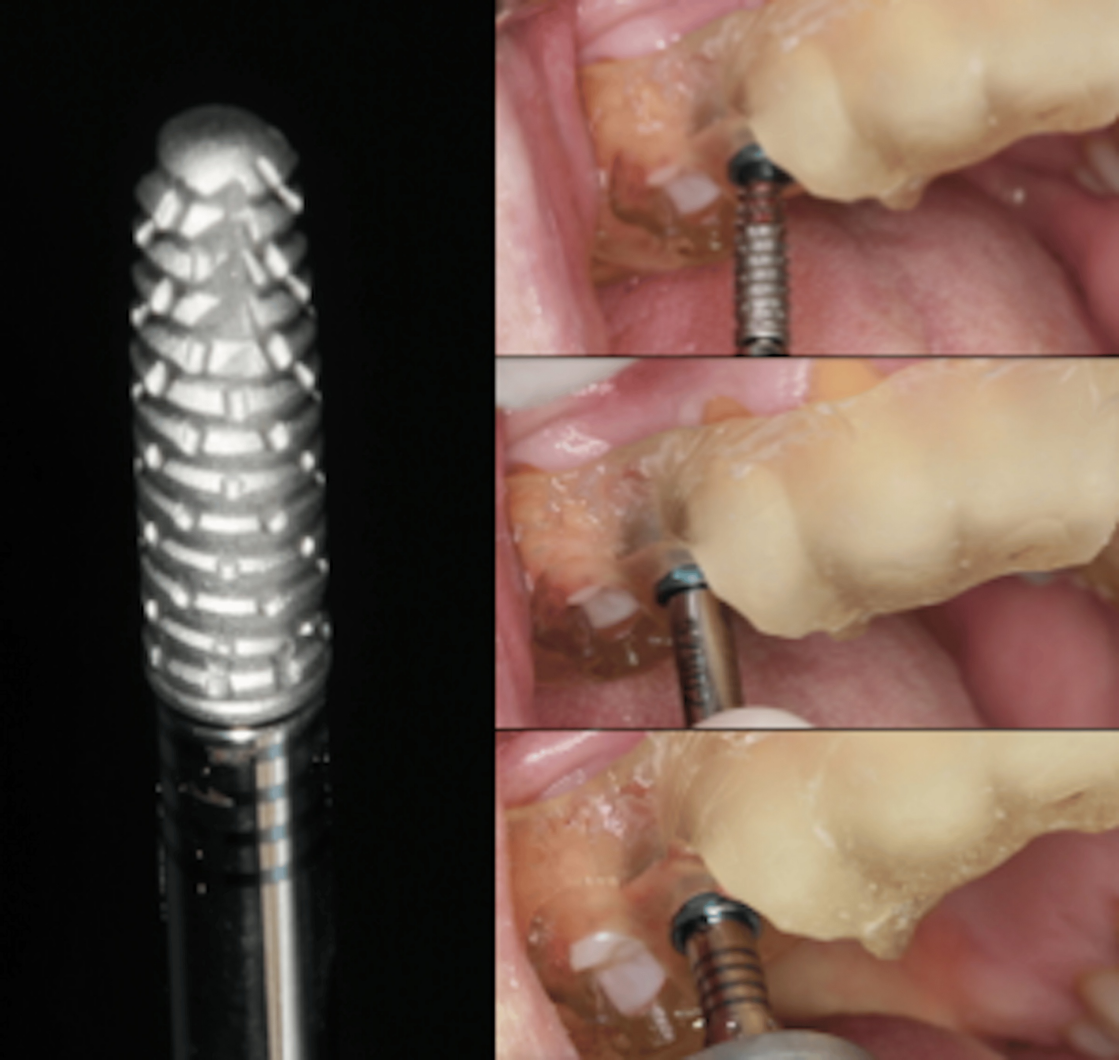
Final implant placement in the region of tooth 15, completed using a ratchet

**Figure 13:**
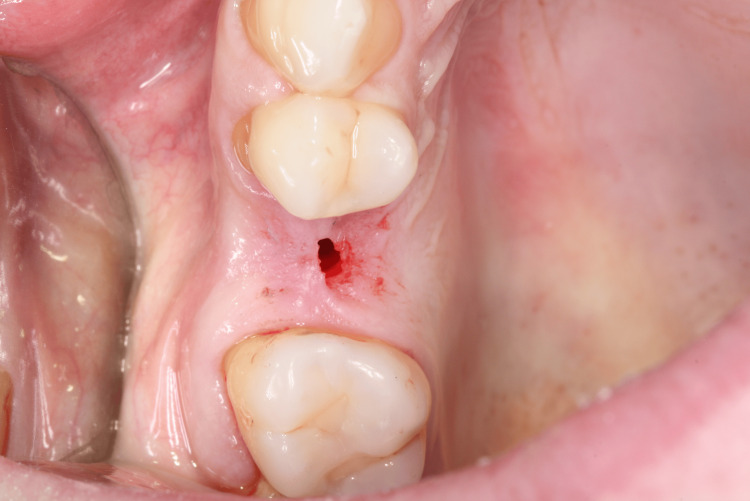
Occlusal view of the installed implant

After positioning the surgical guide (Figure 8, Figure 9), guided instrumentation was performed according to the implant manufacturer’s instructions (Figure 10, Figure 11). This was followed by the placement of the implant, which was conducted with the surgical guide in place (Figure 12, Figure 13).

**Figure 14:**
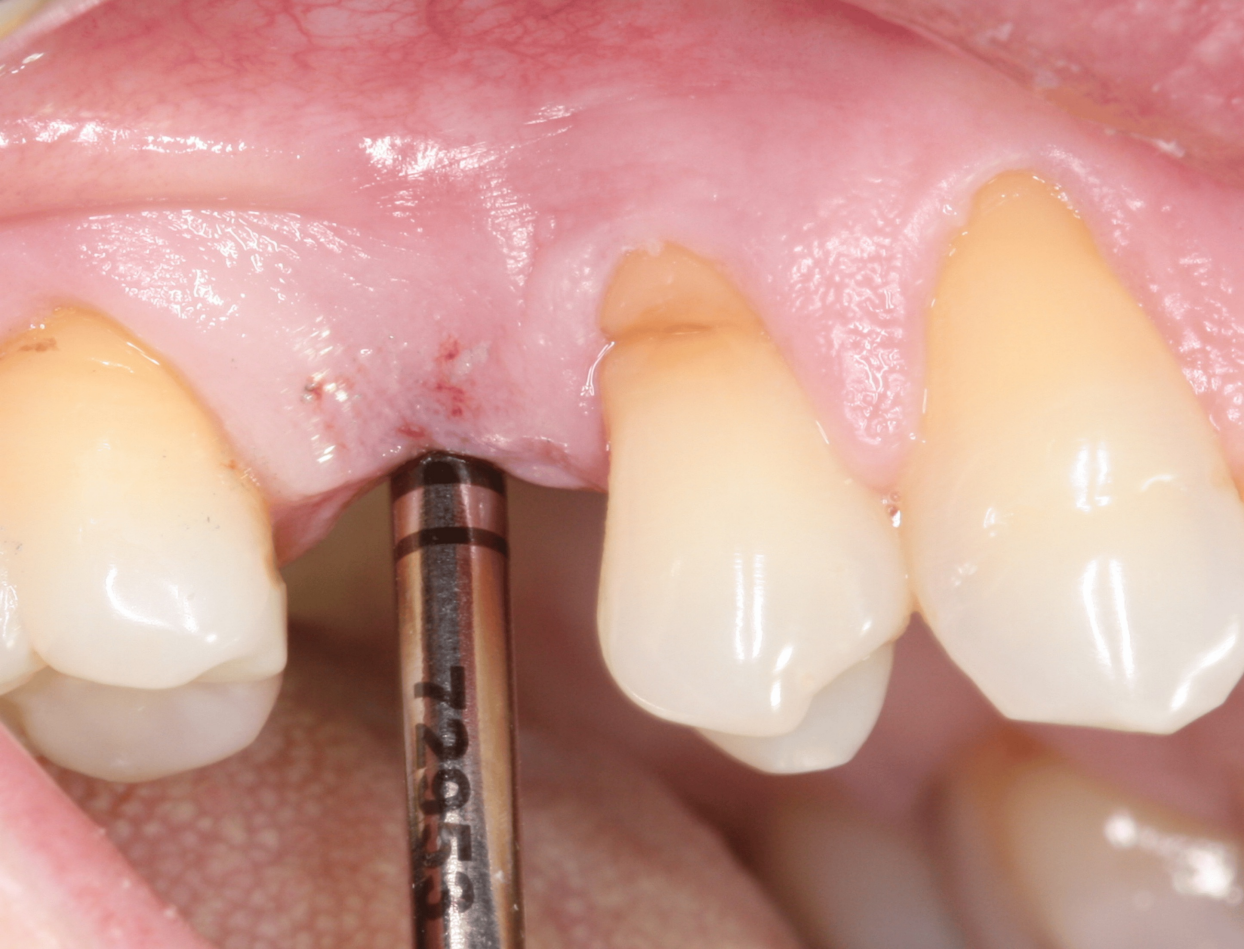
Verification of depth for installing the healing device

**Figure 15:**
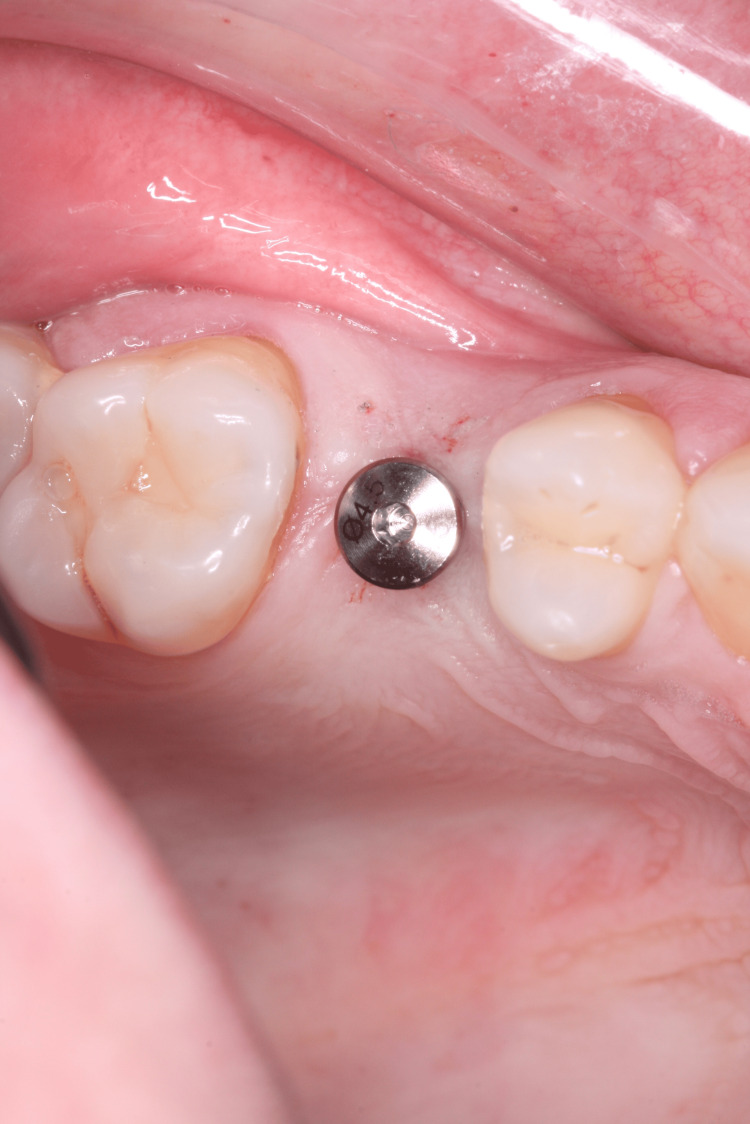
Healing abutment installed immediately after implant placement

**Figure 16:**
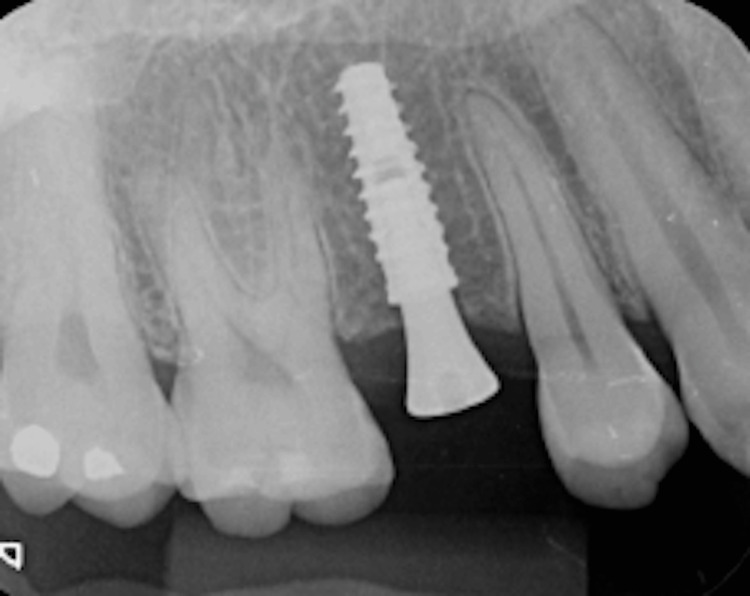
Final radiograph of implant 15 immediately after placement, with the healing abutment in place

For tooth 35, an Implacil UN III Duecone AR implant (3.5 mm × 9 mm) was placed at 1 mm intraosseous (IO). For tooth 15, an Implacil UN III Maestro AR implant (3.5 mm × 11 mm) was placed at 2 mm IO. Healing devices were installed (Figure 14, Figure 15) to avoid a second surgical procedure after the osseointegration period. An X-ray was taken to confirm the implant position (Figure 16).

**Figure 17:**
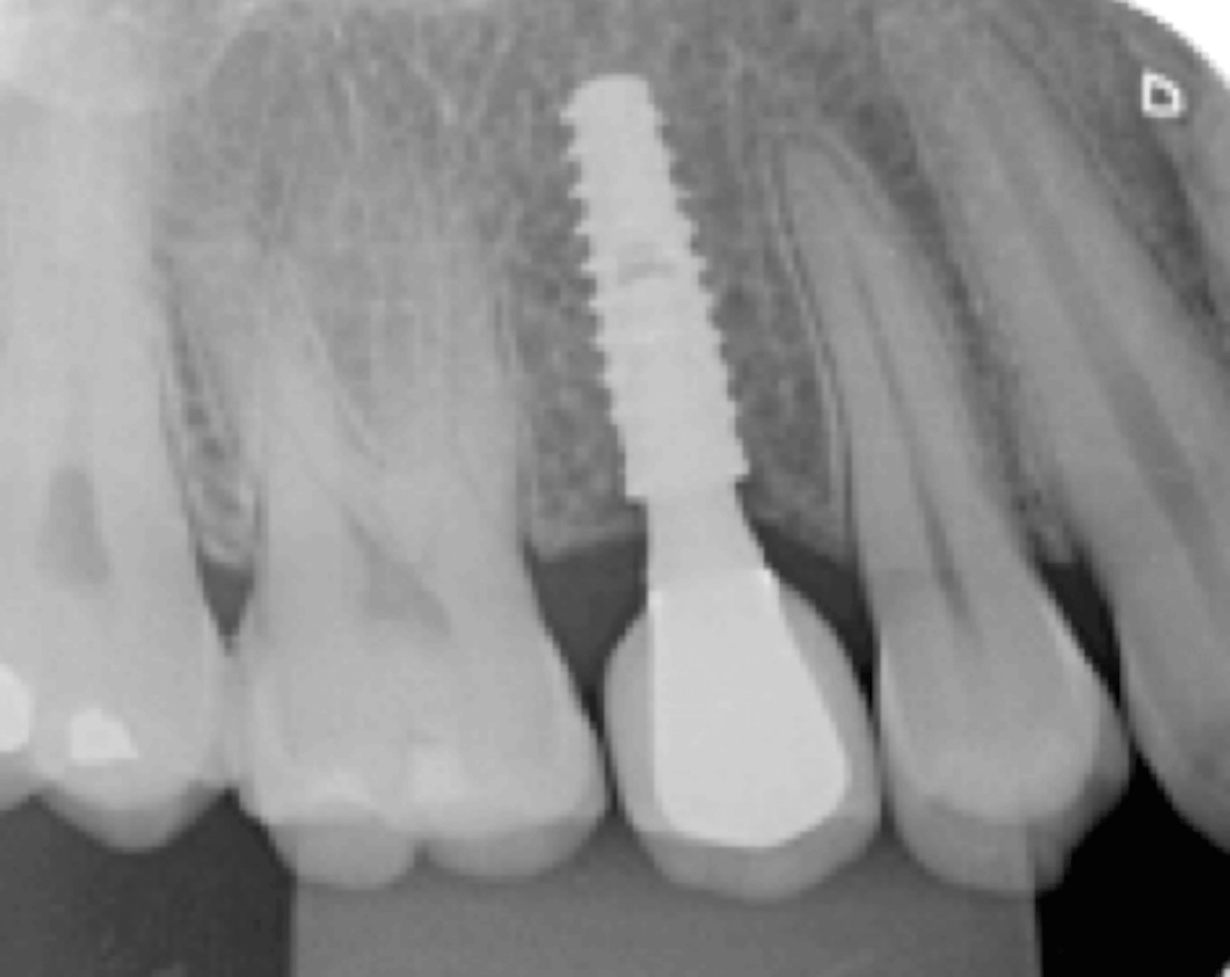
Radiograph showing abutment and provisional restoration installed for implant 15

After four months, the final abutments and temporary crowns were installed (Figure 17). For tooth 35, a 3.3 × 4 × 1.5 mm Ideale Cone Morse Abutment (Implacil de Bortoli, São Paulo) was placed. For tooth 15, a 3.3 × 4 × 2.5 mm Ideale Cone Morse Abutment (Implacil de Bortoli, São Paulo) was installed.

**Figure 18:**
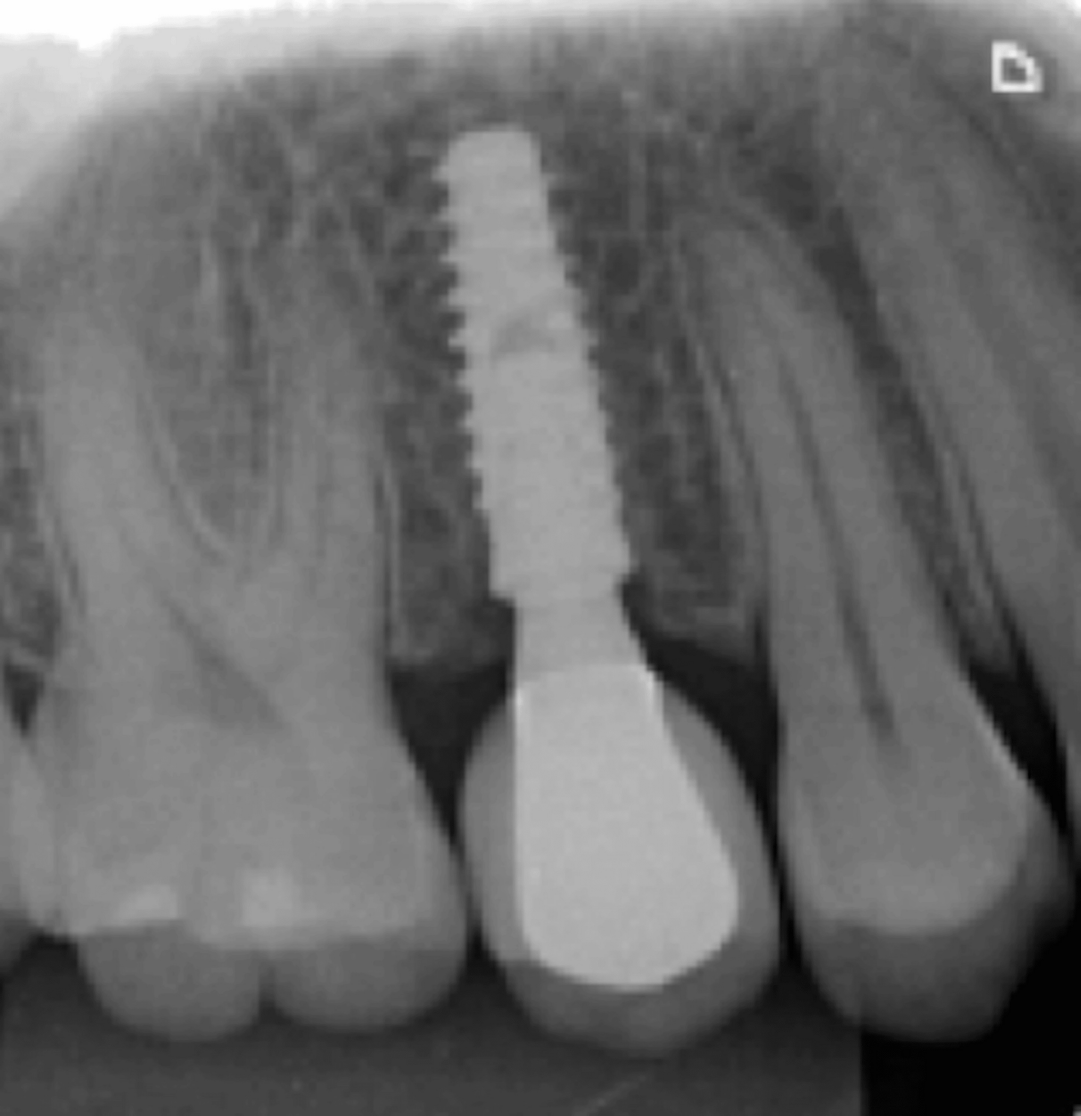
Control radiograph of implant 15 with the ceramic screw-retained crown

**Figure 19:**
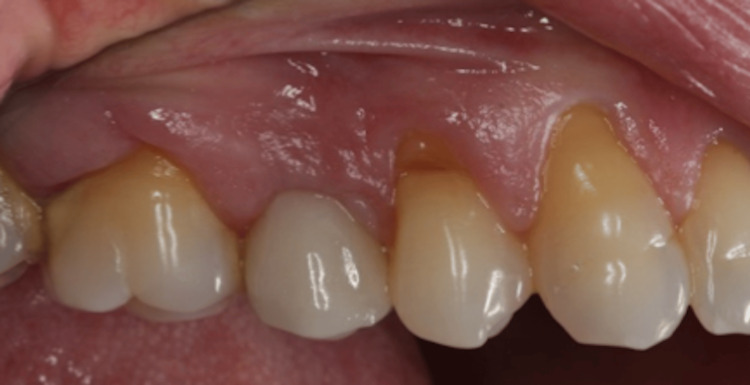
Buccal view of the ceramic crown of implant 15

**Figure 20:**
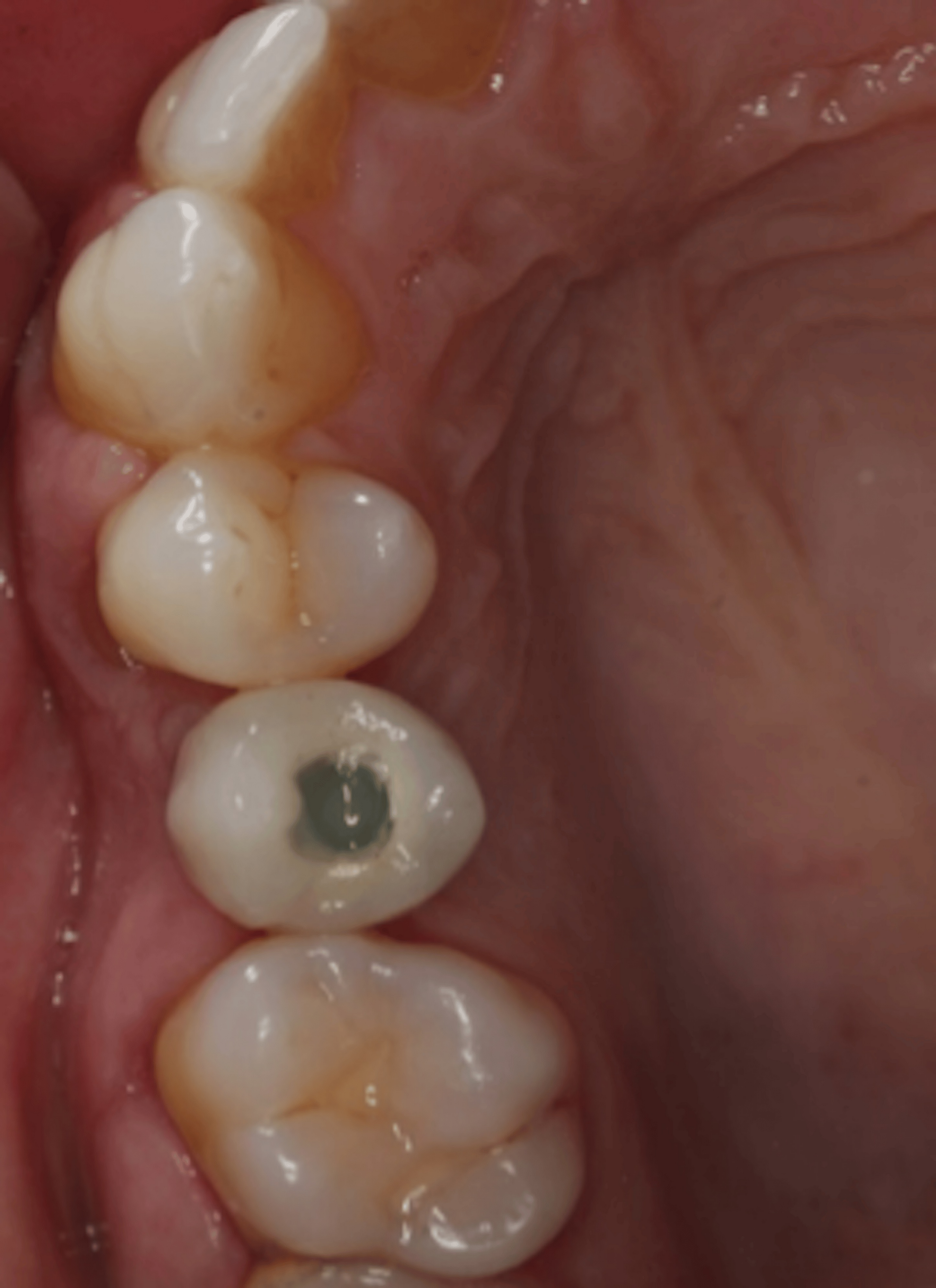
Occlusal view of the screwed ceramic crown on implant 15

During the subsequent appointments, an impression was taken to produce metal-ceramic crowns, and the color was selected. In the following stage, the screwed-retained crowns were installed, and necessary adjustments were made (Figure 18, Figure 19, Figure 20).

## Discussion

Guided surgery offers several advantages over conventional methods, such as the ability to perform surgery without raising flaps. In this case report, guided surgery was chosen due to the patient’s systemic complications, which contraindicated flap procedures [4]. Preoperative tomography scans were crucial for assessing the bone quantity required for implant placement, ensuring satisfactory functional and aesthetic results [6].

The patient in this case had comorbidities and was on anticoagulant medications. According to Cattoni et al., guided surgery is particularly recommended for such patients due to its less traumatic nature, reduced need for analgesics, faster healing, and the ability to resume oral hygiene immediately post-procedure [11].

Studies, including one by Yang et al., support guided surgery for patients with comorbidities. This technique, which uses conical implants in immunosuppressed patients, is preferred for its increased predictability and reduced risk of surgical complications [12].

Although guided surgery requires less experience compared to conventional methods due to its greater predictability, it is important to note that it is contraindicated in cases of restricted mouth opening and insufficient bone volume, highlighting the need for thorough planning [13]. The success of the surgery in this study was attributed to the reliable surgical guides that accurately indicated the ideal implant placement, a finding supported by several studies comparing post-procedure precision with presurgical virtual planning [14,15].

## Conclusions

Guided surgery using advanced software enhances precision in achieving the ideal implant placement. This technique, driven by technological advancements, promotes faster recovery, greater postoperative comfort, and reduced pain and edema.

In this case report, the use of flapless guided surgery was particularly beneficial for a patient with comorbidities. It underscores the importance of detailed patient anamnesis for the successful planning and execution of the procedure by implant dentists.
